# Plasma Homocysteine Levels and Cardiovascular Events in Patients With End-Stage Renal Disease: A Systematic Review

**DOI:** 10.7759/cureus.40357

**Published:** 2023-06-13

**Authors:** Abdulilah A ALSolami, Abdulrhman A Almalki, Saleh Yousef Alhedyan, Abdulmajeed Alghamdi, Sultan M Alzahrani, Wesam R Dause, Fahad A Hamdi, Mohannad T Howladar, Islam A Ibrahim

**Affiliations:** 1 Nephrology, King Fahad Armed Forces Hospital, Jeddah, SAU; 2 Cardiology, King Fahad Armed Forces Hospital, Jeddah, SAU; 3 Cardiology, King Fahad Military Medical Complex, Jeddah, SAU; 4 Cardiology, Ministry of Health, Jeddah, SAU

**Keywords:** nutrition, risk, mortality, cardiovascular disease, homocysteine

## Abstract

Background: Patients with chronic renal disease or failure are at a heightened risk of mortality due to cardiovascular disease (CVD), which is a predominant cause of death in this population. Hyperhomocysteinemia is prevalent in patients with end-stage renal disease (ESRD), which may increase their susceptibility to CVD.

Methods: We conducted a comprehensive search of PubMed, Science Direct, and Google Scholar for articles published between 2003 and February 2023, using a combination of keywords such as "plasma homocysteine levels," "hyperhomocysteinemia," "end-stage renal disease," "renal failure," "kidney failure," "cardiovascular events," "cardiovascular disease," "myocardial infarction," "coronary artery disease," and "stroke." Our inclusion criteria were studies that investigated the association between total homocysteine (Hcy) level and CVD or total mortality, as well as the impact of vitamin supplementation on cardiovascular or mortality risk. We restricted our search to English-language studies that included ESRD patients and provided data on plasma Hcy levels and associated CVD events.

Results: This systematic review includes 11 articles published between 2003 and 2023 that enrolled a total of 3,953 subjects, of whom 79.15% were male patients. All studies included in the review were either quantitative randomized trials or non-randomized studies, such as cross-sectional, cohort, or case-control studies. Of the total studies included, 10 reported either cardiovascular mortality or CVD events, including cardiovascular death, myocardial infarction (MI), angina, and stroke. One study reported the CVD risk score of the patients, and most of them had higher total homocysteine (tHcy) levels. Overall, a total of 817 CVD events were reported across the studies.

Conclusion: In conclusion, the relationship between Hcy and cardiovascular events in ESRD patients is not straightforward and requires further research. However, our review suggests that Hcy could be a predictor of cardiovascular events in this population, and its nutritional characteristics as well as other associated comorbidities may contribute to its inverse association with outcomes.

## Introduction and background

Homocysteine (Hcy) is a sulfur-containing non-essential amino acid that plays a significant role in the Hcy-methionine cycle through its interaction with folic acid and vitamin B12. Disruption of this metabolic process may result in Hcy buildup, which can lead to clinically significant outcomes such as calcified vascular tissues, atherothrombosis, cardiac vasculopathy, and impaired cognition [1]. It is hypothesized that even a modest rise in total Hcy concentration may result in cardiovascular disease (CVD). Patients with highly elevated levels of total Hcy (>100 mol/L) due to congenital metabolic failings impacting cystathionine-β-synthase have been observed to experience sharply advancing atherosclerotic and thromboembolic events [2].

Increased susceptibility to cardiovascular events is a major reason for the elevated mortality rate in patients with end-stage renal disease (ESRD). The life expectancy of individuals with ESRD is significantly lower than those with normal kidney function, estimated to be less than 50% [3]. When compared to the general population, individuals with ESRD have higher rates of mortality and require dialysis [4]. Contributing factors to this include underlying renal disease, pre-existing coronary artery disease, nutritional deficiencies, systemic inflammation, and anaemia [5,6]. Hcy is considered an unconventional prognostic biomarker for CVD in both the general population and individuals with chronic kidney disease (CKD) [3].

In patients with normal kidney function, hyperhomocysteinemia is recognized as a separate risk factor for atherosclerosis. However, due to abnormal renal processing and reduced urinary clearance, hyperhomocysteinemia affects approximately 85% of individuals with CKD [7,8]. Individuals who have chronic kidney failure have higher plasma Hcy concentrations, which may contribute to their significant CVD-related morbidity and mortality. In ESRD patients without diabetes, successful correction of plasma Hcy concentrations may lead to a reduction in CVD-related morbidity and mortality. However, it is important to note that in ESRD patients, other factors may also affect the efficacy of Hcy-lowering medications, including genetic polymorphisms, consumption of fortified grains, inflammatory processes, malnutrition, and high blood glucose levels. Gender, genetics, diet, and required supplementation within different communities may also impact how individuals respond to Hcy-lowering medication. Moreover, diabetic ESRD patients who exhibit treatment resistance may require alternative therapeutic approaches. While the average total Hcy level in the general population ranges from 10 to 15 μmol/L depending on age, gender, and location, it is typically 25 to 35 μmol/L in ESRD patients [9,10], making them the second-highest group after patients with homocystinuria in terms of total Hcy levels [11]. While total Hcy level has been linked to CVD and related deaths in the overall population in several meta-analyses, no such systematic review has been done for individuals with ESRD.

The potential benefits of Hcy-lowering therapy in ESRD patients are still being investigated. Some studies have suggested that Hcy-lowering therapy may be effective in reducing CVD-related morbidity and mortality in ESRD patients [12], while others have reported conflicting results [12-14]. Nevertheless, because individuals with ESRD have substantially higher concentrations of total Hcy, findings from prospective observational studies and RCTs cannot be readily generalized to these patients. Consequently, it is unclear if total Hcy levels are significant as a cardiovascular and mortality risk factor in the case of ESRD. Even though patients with ESRD have been the focus of several investigations using a variety of study designs, the findings are currently neither consistent nor conclusive [15]. The present systematic review aims to investigate the association between plasma Hcy levels and cardiovascular events in patients with ESRD.

## Review

Materials and methods

Definition of Outcomes and Inclusion Criteria

The primary outcome of this systematic review was nonfatal and fatal cardiovascular events or CVD as well as all-cause mortality in studies of patients with ESRD. The basic inclusion criteria were patients with ESRD. Additional inclusion criteria were as follows: cases were dialysis patients with ESRD diagnosed by using established methods at the time of publication; outcomes were total mortality and cardiovascular morbidity; measurement of blood tHcy by using established laboratory methods; and availability of data for patients’ baseline data. Non-original studies, case studies (less than five cases), incomplete studies, abstract-only articles, protocols, reviews, and conference proceedings were screened to exclude those that did not meet the inclusion criteria. Primary literature would be specific only for ESRD patients and Hcy levels and cardiovascular events. Also, studies evaluating the influence of folate treatment or vitamin supplementation on cardiovascular or mortality risk perspectives were included in the systematic review.

Search Strategy

We conducted a comprehensive search of the PubMed, Google Scholar, and Science Direct databases, covering the timeline from 2003 to 2023. We used keywords such as "plasma homocysteine levels," "homocysteine," "homocyst(e)ine," "hyperhomocysteinemia," "end-stage renal disease," "renal failure," "kidney failure," and "cardiovascular events," "cardiovascular disease," "myocardial infarction," "coronary artery disease," and "stroke" to search all the relevant databases. No restrictions were applied for country, ethnicity, or gender. To ensure the inclusion of only relevant studies, we restricted our search strategy to the title and abstract of the search results. After collecting these results, we exported them to an Endnote library and identified and removed duplicate studies that appeared across multiple searched databases. Reference sections and annotated bibliographies of included studies were cross-referenced for additional eligible studies. The entire process of this systematic review was carried out in accordance with the guidelines of the Preferred Reporting Items for Systematic Reviews and Meta-Analyses (PRISMA) [16].

Screening and Extraction

To ensure accuracy in the screening process, we implemented a double screening strategy that involved reviewing both the titles and abstracts as well as the full texts of potential studies. Once we confirmed that all relevant articles were included, we developed a data collection sheet that was organized according to our desired outcomes and included baseline characteristics and sought outcomes. For each study, data on study characteristics (including the year of publication, country of origin, design, sample size, gender distribution, and study follow-up), Hcy level, and cardiovascular events and comorbidities were extracted and entered into pre-built tables.

Quality Assessment

According to the PRISMA statement, the evaluation of methodological quality provides an indication of the strength of the evidence included in the study because methodological flaws can result in biases. Due to the wide range of types of studies finally included in this systematic review, we decided to use the Mixed Methods Appraisal Tool (MMAT) [17]. This tool was based on the following criteria: appropriateness of the mixed methods design, adequacy of the sampling strategy, quality of data collection, quality of data analysis, and integration of the qualitative and quantitative components. Each criterion was assessed using a series of questions and scoring criteria. The possible responses to all questions were "yes," "no," or "can't tell." The response "no" to two of the screening questions or "can't tell" to one or both of the screening questions might indicate that the paper cannot be appraised using the MMAT. Positive responses indicate the high quality of the evidence presented in the study, while "can't tell" indicates a failure to report exact results that meet the assumptions of the question.

Results

Search Results

**Figure 1:**
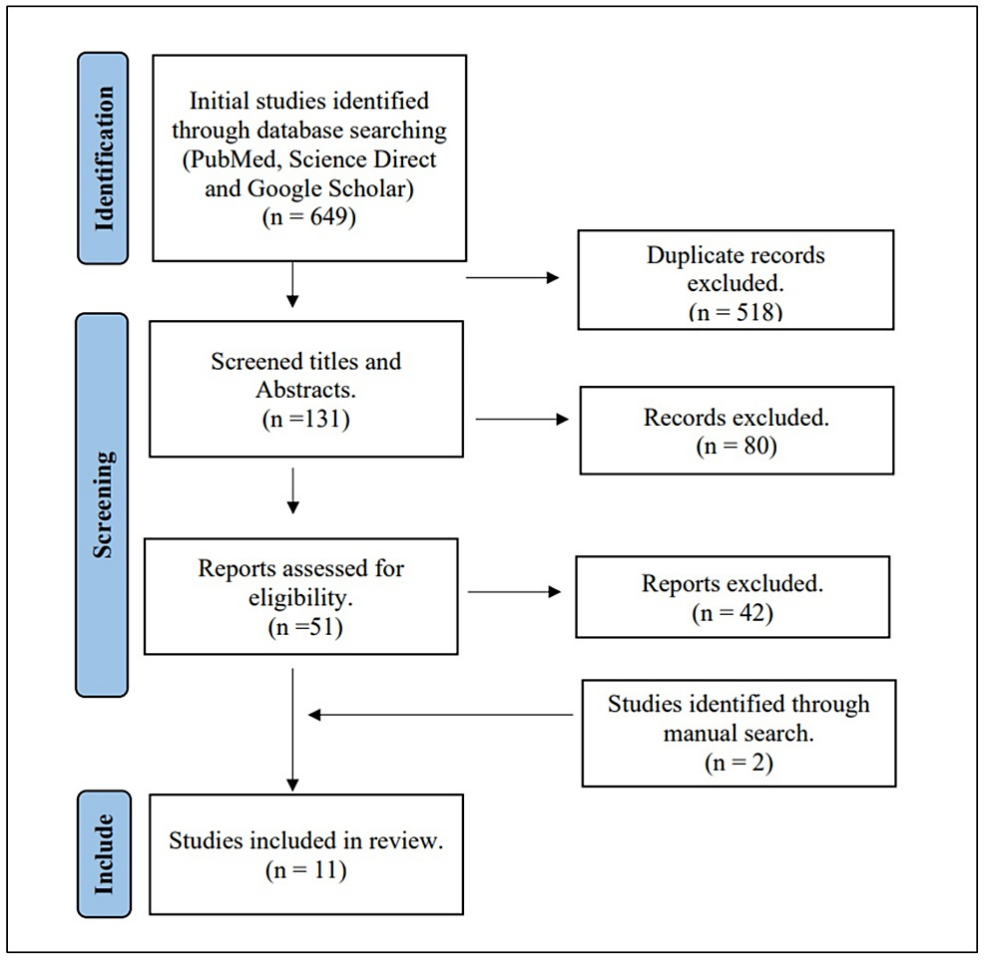
PRISMA flow chart PRISMA: Preferred Reporting Items for Systematic Reviews and Meta-Analyses

A total of 649 abstracts were screened from the 132 publications obtained using the aforementioned search strategy, with English as the only language. If the abstracts were found relevant, the full-text article was reviewed. Of the 51 articles assessed, nine met all of the criteria for inclusion and were included and investigated in the review; also, two articles were found through reference searches. A total of 11 studies with a total of 3953 patients fulfilled the inclusion criteria and are presented in the analysis (Figure 1).

Results of Quality Assessment

For quantitative randomized controlled trials (RCTs), most of the studies appear to meet the criteria for clear research questions and data that address those questions. However, adherence to the intervention was unclear in some studies. Additionally, all studies classified as low-risk studies met 75-100% of the MMAT criteria, indicating high-quality evidence (Table 1).

**Table 1:** Quality assessment of the studies included in the systematic review using Mixed Methods Appraisal Tool (MMAT) version 2018.

	Screening Questions		Quantitative Randomized Controlled Trials				
Study Authors	Are there clear research questions?	Do the collected data address the research questions?	Is randomization appropriately performed?	Are the groups comparable at baseline?	Are there complete outcome data?	Are outcome assessors blinded to the intervention provided?	Did the participants adhere to the assigned intervention?
Righetti et al. [18]	Yes	Yes	Yes	Yes	Yes	Yes	Can’t tell
Righetti et al. [19]	Yes	Yes	Yes	Yes	Yes	Yes	Can’t tell
Thaha et al. [20]	Yes	Yes	Yes	Yes	Yes	No	Can’t tell
Jamison et al. [21]	Yes	Yes	Yes	Yes	Yes	Yes	Yes
Heinz et al. [22]	Yes	Yes	Yes	Yes	Yes	Yes	Yes
			Quantitative Nonrandomized Study				
	Are there clear research questions?	Do the collected data address the research questions?	Are the participants’ representative of the target population?	Are measurements appropriate regarding both the outcome and intervention (or exposure)?	Are there complete outcome data?	Are the confounders accounted for in the design and analysis?	During the study period, is the intervention administered (or exposure occurred) as intended?
Buccianti et al. [23]	Yes	Yes	Yes	Yes	Yes	Yes	No
Bayés et al. [24]	Yes	Yes	Yes	Yes	Yes	Yes	Yes
Kalantar-Zahed et al. [25]	Yes	Yes	Yes	Yes	Yes	No	No
Pernod et al. [26]	Yes	Yes	Yes	Yes	Yes	Yes	Yes
Ganeshan et al. [27]	Yes	Yes	Yes	Yes	Yes	No	Yes
Sandeep et al. [28]	Yes	Yes	Yes	Yes	Yes	No	Yes

Characteristics of the Study Included

This systematic review looks at 11 articles that were taken from 2003 to 2023 and enrolled a total of 3953 subjects, of whom 79.15% were male patients. All the included studies were either quantitative randomized trials or non-randomized studies such as cross-sectional, cohort, or case-control studies. Italy was the setting for three studies, while India and the USA were represented in two studies each. Spain, Indonesia, France, and Germany were each represented in a single study. The included studies in the review had a follow-up period ranging from two to six years. Table 2 displays all the baseline characteristics of these studies.

**Table 2:** Baseline characteristics of the included studies RCT: randomized controlled trial; ESRD: end-stage renal disease; NR: not reported

Author	Country	Study Type	Year of Publication	Total Sample Size	Age (in years)	Sex M/F	Follow up
				Case/control			
Buccianti et al. [23]	Italy	observational	2003	77	62.85 ± 1.53	47/30	44 months
Bayés et al. [24]	Spain	observational	2003	94	66 ± 14	50/44	24 months
Righetti et al. [18]	Italy	RCT	2003	30/51	64 ± 3	45/36	12 months
Zadeh et al. [25]	USA	observational	2004	367	54.5 ± 14.7	199/168	12 months
Righetti et al. [19]	Italy	RCT	2006	63/51	A = 64.0 ± 82.3, B = 63.9 ± 81.6 C = 65.18 ± 1.9	63/51	871 days
Thaha et al. [20]	Indonesia	RCT	2006	30/30	treatment = 48.10 ± 11.08, placebo = 52.60 ± 10.30	39/21	NR
Pernod et al. [26]	France	observational	2006	279	63.77 ± 13.7	179/100	2 years
Jamison et al. [21]	USA	RCT	2007	1032/1024	treatment: 65.4 (12.0), Placebo: 66.2 (11.5)	2023/33	38 months
Heinz et al. [22]	Germany	RCT	2010	327/323	61 ± 13	379/271	6 years
Ganeshan et al. [27]	India	observational	2014	50/25	case = 52.9 ± 4.69, control = 49 ± 2.404	49/26	NR
Sandeep et al. [28]	India	observational	2018	75/25	ESRD = 55 (49.5-65.0), control = 50 (43.5-57.0)	56/44	NR

Of the total included studies, 10 reported CVD events, including cardiovascular death, myocardial infarction (MI), angina, and stroke. One study reported the CVD risk score of the patients [28], and most of them had higher tHcy levels. A total of 817 CVD events were reported. All-cause mortality of the included study patients was reported in five studies. Of the total studies, five examined the impact of vitamin B therapy on CVD in ESRD patients by lowering Hcy levels [18,19,21,22,27], while one study investigated the effects of intravenous N-acetylcysteine during haemodialysis on the Hcy plasma concentration in ESRD patients [20]. All RCT and case-control studies, except for the one with a large sample size [22], supported folate supplementation for Hcy levels and vascular disease in ESRD patients. However, Heinz et al. [22] did not endorse increased intake of folic acid, vitamin B12, and vitamin B6, as it did not lower total mortality or significantly impact the risk of cardiovascular events in patients with ESRD. Interestingly, one study revealed an unexpected association, where lower values of tHcy were linked to increased hospitalization and mortality in patients (Table 3) [25].

**Table 3:** Summary of the outcomes of the included studies in this review. NR: not reported; CVD: cardiovascular disease; ESRD: end-stage renal disease; MI: myocardial infarction; HTN: hypertension; DM: diabetes mellitus

Author	Dialysis Mode	Homocysteine (Hcy) Concentration (μmol/L)	Cardiovascular Outcome (Mortality or CVD)	Other Complications/Comorbidity	Follow up
Buccianti et al. [23]	Hemodialysis	--	CVD deaths, 20/77 (Hcy Q4 (12), Q3 (2), Q2 (4) Q1 (2))	--	44 months
Bayés et al. [24]	Hemodialysis	Mean plasma Hcy = 25.8 (7.8). Patients with normal Hcy (<15) = 5.7%	Total deaths = 32 CVD deaths = 19/32 non-CVD death = 13/32	Inflammation, mean serum CRP = 11 (16) mg/l Patients with CRP > 6 mg/l = 45.3%	24 months
Righetti et al. [18]	Hemodialysis	Treated vs. untreated; 5mg FA=47.2±7.1, 15 mg FA = 53.2±7.4, Untreated = 50.5±6.1	Nonfatal CVD: control, 11/30 (36) treatment, 13/51 (25)	DM, New vascular events during follow-up in diabetics vs. non-diabetics = 44% vs. 25%	12 months
Zadeh et al. [25]	Hemodialysis	All: 1.35 ± 0.17, surviving: 1.36 ± 0.16, deceased: 1.28 ± 0.19	All-cause Mortality: 37/367, CVD deaths: 26/367, CVD deaths, tHcy: Q1: 10.9%, Q2: 6.5%, Q3: 5.4%, Q4: 5.4%	DM, History of CVD	12 months
Righetti et al. [19]	Hemodialysis	Treatment A, B= 21.88 ± 1.4, 37.28 ± 1.2, control = 32.78 ±1.6	All-cause mortality = 25, CVD deaths: 14, Non-fatal CVD events: treatment = 19, control = 25	DM	871 days
Thaha et al. [20]	Hemodialysis	Placebo (from 24.5 ± 11.7 to 18.7 ± 9.2); N-Acy = 88.3% (from 28.1 ± 21.1 to 3.3 ± 3.3)	Pulse pressure decrease, Placebo = 84.0 vs 91.1mm; N-Acy = 82.0 vs. 67.0mm Mortality, 0/60; nonfatal CVD = 0/60	Hypotension, Placebo group = 0/30, N-Acy = 2/30; Urticaria, Placebo group = 0/30, N-Acy = 1/30	NR
Pernod et al. [26]	Hemodialysis vs peritoneal dialysis = 244 vs 35	tHcy >14 = 245/279 tHcy < 30 vs tHcy ≥30 = 157 (60.1%) vs 104 (39.8%)	All-cause mortality= 54, Total CVD events = 82 CVD deaths = 26	NR	2 years
Jamsion et al. [21]	Hemodialysis	Placebo = 22.3 (18.7-26.9), treatment = 22.5 (18.9-27.3)	Mortality: mortality placebo group = 436, vitamin group deaths = 448, CVD events: placebo = 191, Vitamin = 166	History of MI, HF, HTN, Angina, stroke, and DM	38
Heinz et al. [22]	NR	Placebo: 28.2 (13.0–62.0), treatment: 30.0 (14.9–65.3)	Cardiovascular events: placebo: 98 (30), treatment: 83 (25)	DM, HTN, history of CVD	6 years
Ganeshan et al. [27]	NR	Control: 14.4 ± 1.39 Case: 22.91± 3.125	Angina = 36 (72), acute AMI = 9 (18), stroke = 5 (10)	HTN, Hyperlipidemia	NR
Sandeep et al. [28]	NR	Control: 12.1 (9.1-16), ESRD: 19.9 (17.1-23.5)	CVD risk: case: 2.0 (-0.5-4.0), Control: 2.0 (-0.5-4.0)	DM, HTN	NR

Discussion

ESRD is a severe condition that carries a high risk of all-cause mortality and CVD. Identifying biomarkers that can predict CVD development in ESRD patients is a crucial goal in clinical practice. Hyperhomocysteinemia is prevalent in ESRD patients and can increase their susceptibility to CVD [29]. Given that CVD is the major cause of mortality in ESRD patients, it is important to identify and evaluate factors that lead to their increased CVD risk. Hcy levels have been proposed as a potential biomarker for CVD risk in ESRD patients. This systematic review aimed to evaluate the relationship between plasma Hcy levels and cardiovascular events in ESRD patients.

The study included in this review presented mixed findings; however, despite the limitations of the studies and the use of folate supplements, the current systematic review demonstrated that there is a connection between high plasma Hcy levels and an increased risk of all-cause mortality and cardiovascular events in ESRD patients. These results are consistent with previous research that has found similar associations between Hcy and CVD in other patient populations, including those with hypertension or diabetes, as well as in the general population.

The relationship between Hcy and cardiovascular events in ESRD patients is complex and multifactorial. Hcy has been demonstrated to induce oxidative stress, inflammation, and endothelial dysfunction, all of which are associated with the pathogenesis of CVD [30]. Furthermore, Hcy can impair the function of vascular smooth muscle cells, promote the development of atherosclerotic plaque, and increase the risk of thrombosis [31]. These effects could contribute to the higher risk of CVD observed in ESRD patients with elevated plasma Hcy levels.

Elevated serum tHcy levels are prevalent in the chronic renal failure or ESRD population [29]. Hcy has a short intracellular half-life and is efficiently exported into the extracellular medium, with an increase in tHcy being the first indicator of CVD. Patients with ESRD often have persistent, mild hyperhomocysteinemia. Previous studies, not included in this review, have demonstrated that hyperhomocysteinemia is linked to an increased risk of CVD, which was confirmed by later studies such as Buccianti et al. [23], who discovered that Hcy is a strong independent predictor of mortality in hemodialysis patients. Patients with higher total plasma Hcy levels also have a higher left ventricular mass index, which is associated with a greater incidence of heart failure [32,33]. Irena et al. [34] concluded that Hcy and other laboratory parameters are the preferred laboratory markers for evaluating the risk of heart attack or stroke in dialysis patients. The diagnostic value of certain parameters, such as cholesterol, low-density lipoprotein (LDL), triglycerides, and albumin, was also found to be significant [34]. Existing data suggest that Hcy has direct effects on the myocardium, as well as nitrous oxide-independent vascular effects [35]. Sandeep et al. [28] developed a score to assess the overall risk associated with CKD, which includes new markers of CVD risk. The use of statins in CKD management led to a decrease in lipid profile parameters, while new risk factors such as Hcy and MDA increased with disease progression. Pernod et al. [26] in their observational study propose that ESRD patients who initiate chronic dialysis with a history of cardiovascular events, elevated total Hcy levels, or possessing the MTHFR 677TT genotype should be classified as having a heightened likelihood of experiencing future cardiovascular events. According to an RCT conducted by Taha et al. [20], intravenous administration of N-acetylcysteine during hemodialysis normalized plasma Hcy concentration, resulting in improved pulse pressure in patients with end-stage renal failure.

Several meta-analyses have examined the potential cardiovascular benefits of Hcy-lowering therapies in CKD patients due to the importance of reducing mortality and cardiovascular events, as well as the conflicting results reported in clinical studies. Heinz et al. conducted a meta-analysis of observational studies and RCTs to evaluate the effect of Hcy on overall mortality and the risk of CVD. Their findings indicate that elevated levels of Hcy increase the risk of cardiovascular events and overall mortality. However, supplementation with vitamin B was found to significantly reduce the risk of CVD without a concomitant decrease in overall mortality [36]. However, the study had limitations, such as the small number of analyzed RCTs, and some studies had small sample sizes or non-randomized protocols. A meta-analysis of 4,836 CKD patients also found no significant reduction in CVD, stroke, or all-cause mortality with Hcy-lowering therapy [37]. Nigwekar et al. included 2,452 hemodialysis patients and found no statistically significant difference in CVD events between patients who were treated with supplements aimed at lowering Hcy levels and those who were not [38]. Previous clinical trials and meta-analyses have investigated various treatments that are known to lower Hcy levels, including folic acid, B vitamins, N-acetylcysteine, and omega-3 fatty acids [39]. However, it remains unclear whether these therapies can effectively reduce cardiovascular events in patients. The conflicting findings from these studies suggest that the association between plasma Hcy levels and CVD or mortality in ESRD patients undergoing hemodialysis is still uncertain. While some studies have found a significant association between high Hcy levels and an increased risk of CVD or mortality, others have found no association or even a reverse association between low Hcy levels and worse outcomes.

The inconsistent results in the association between Hcy levels and CVD or mortality in ESRD patients undergoing dialysis may be attributed to deficiencies in water-soluble vitamins such as folate and vitamin B, which are essential for Hcy metabolism. Patients with ESRD undergoing dialysis may be more susceptible to vitamin deficiencies due to the removal of water-soluble vitamins during dialysis. Thus, low Hcy levels in these patients may reflect malnutrition and insufficient vitamin intake, which could contribute to worse outcomes.

Multiple clinical trials have investigated the impact of folic acid and B vitamins on reducing tHcy levels in patients with renal disease. In a short-term study of uremic patients, two years of folic acid supplementation normalized tHcy blood levels in 92.3% of patients but did not change the incidence of cardiovascular events compared to the control group [40]. However, ultrasonography of the common carotid arteries conducted at the beginning and end of a 24-month study revealed a notable decrease in intima-media thickness as a result of folate supplementation [40]. A double-blind, randomized, and placebo-controlled trial conducted over two years with 186 ESRD patients who were administered 10 mg of folic acid three times a week after each dialysis session found that this treatment led to the normalization of Hcy concentrations in 92.3% of patients and, a significant reduction in intima-media thickness. However, there were no observable changes in cardiovascular events in comparison to the control group [22]. In contrast to the previously mentioned study, Righetti et al. [18,19] found evidence that folate treatment can decrease cardiovascular events in dialysis patients by reducing Hcy levels. In their first study, they found that high-dose folic acid supplementation did not improve hyper-Hcy lowering efficacy obtained with 5 mg of oral folic acid daily, and total Hcy-reduction treatment probably reduces the risk for arteriosclerotic vascular disease. In their second study, they found that folate therapy, which reduces Hcy levels, can lead to a decrease in cardiovascular events in patients undergoing dialysis. It is worth noting that the patients in the included studies for this review had diabetes, which is a significant comorbidity. It is important to note that Righetti et al. [19] found a significantly higher mortality rate in diabetic patients despite lower Hcy levels, possibly due to decreased activity of the demethylation pathway in the liver [19]. Therefore, it is recommended that data on Hcy metabolism be presented separately for diabetic and non-diabetic patients. Additionally, Heinz et al. [22] did not support the use of high-dose vitamin supplements for all patients to reduce cardiovascular events, as their study showed no significant effect of folic acid, vitamin B12, and vitamin B6 on mortality and cardiovascular risk in ESRD patients. Indeed, further studies are necessary to establish a clear link between Hcy levels and outcomes in ESRD patients undergoing hemodialysis. Nevertheless, clinicians should still take into consideration the potential impact of Hcy on cardiovascular risk and monitor patients' Hcy levels and nutritional status. Appropriate vitamin supplementation may also be necessary for patients with deficiencies to prevent further complications.

The present systematic review has several limitations. First, the studies were heterogeneous in study design, including randomized and non-randomized quantitative studies. Secondly, small-scale, single-centre studies may have been subject to patient selection and treatment bias. Additionally, the studies had different follow-up periods, which may affect the accuracy of the results. Moreover, certain intervention studies involved treatments aimed at lowering Hcy levels with different endpoints.

## Conclusions

In conclusion, the relationship between Hcy and cardiovascular events in ESRD patients is not straightforward and requires more research. However, our review indicates that Hcy could be a predictor of cardiovascular events in this population, and its nutritional characteristics as well as other associated comorbidities may contribute to its inverse association with outcomes.
